# Polymicrobial synergy stimulates *Porphyromonas gingivalis* survival and gingipain expression in a multi-species subgingival community

**DOI:** 10.1186/s12903-021-01971-9

**Published:** 2021-12-15

**Authors:** Julia R. Davies, Trupti Kad, Jessica Neilands, Bertil Kinnby, Zdenka Prgomet, Torbjörn Bengtsson, Hazem Khalaf, Gunnel Svensäter

**Affiliations:** 1 Section for Oral Biology and Pathology, Faculty of Odontology and Biofilms Research Center for Biointerfaces, Malmö University, 20506 Malmö, Sweden; 2 School of Medical Sciences, Örebro University, Örebro, Sweden

**Keywords:** Microbial community, Periodontitis, Dysbiosis, Virulence, Proteolytic activity

## Abstract

**Background:**

Dysbiosis in subgingival microbial communities, resulting from increased inflammatory transudate from the gingival tissues, is an important factor in initiation and development of periodontitis. Dysbiotic communities are characterized by increased numbers of bacteria that exploit the serum-like transudate for nutrients, giving rise to a proteolytic community phenotype. Here we investigate the contribution of interactions between members of a sub-gingival community to survival and development of virulence in a serum environment—modelling that in the subgingival pocket.

**Methods:**

Growth and proteolytic activity of three *Porphyromonas gingivalis* strains in nutrient broth or a serum environment were assessed using A_600_ and a fluorescent protease substrate, respectively. Adherence of *P. gingivalis* strains to serum-coated surfaces was studied with confocal microscopy and 2D-gel electrophoresis of bacterial supernatants used to investigate extracellular proteins. A model multi-species sub-gingival community containing *Fusobacterium nucleatum, Streptococcus constellatus*, *Parvimonas micra* with wild type or isogenic mutants *of P. gingivalis* was then created and growth and proteolytic activity in serum assessed as above. Community composition over time was monitored using culture techniques and qPCR.

**Results:**

The *P. gingivalis* strains showed different growth rates in nutrient broth related to the level of proteolytic activity (largely gingipains) in the cultures. Despite being able to adhere to serum-coated surfaces, none of the strains was able to grow alone in a serum environment. Together in the subgingival consortium however, all the included species were able to grow in the serum environment and the community adopted a proteolytic phenotype. Inclusion of *P. gingivalis* strains lacking gingipains in the consortium revealed that community growth was facilitated by Rgp gingipain from *P. gingivalis.*

**Conclusions:**

In the multi-species consortium, growth was facilitated by the wild-type and Rgp-expressing strains of *P. gingivalis,* suggesting that Rgp is involved in delivery of nutrients to the whole community through degradation of complex protein substrates in serum. Whereas they are constitutively expressed by *P. gingivalis* in nutrient broth, gingipain expression in the model periodontal pocket environment (serum) appeared to be orchestrated through signaling to *P. gingivalis* from other members of the community, a phenomenon which then promoted growth of the whole community.

**Supplementary Information:**

The online version contains supplementary material available at 10.1186/s12903-021-01971-9.

## Background

The human oral cavity is a highly complex ecosystem in which the tooth- and soft-tissue surfaces offer distinct ecological niches for microbial colonization. Reflecting this habitat diversity, the oral microbiome has been shown to comprise at least 600 different bacterial species [1], of which up to 300 can be found in a single individual [2]. In health, microbial communities exist in a homeostatic balance with the host that contributes to ecosystem stability and provides resistance to colonization by exogenous pathogens. In periodontitis, however, the development of periodontal pockets as a result of breakdown of the bone and soft-tissues supporting the teeth, creates a new subgingival niche that promotes growth of bacterial species suited to the environment. When the adjacent gingival soft-tissues are inflamed, the flow of serum-like transudate (gingival crevicular fluid, GCF) into the pocket increases and under the influence of this environmental perturbation, homeostasis in the subgingival microbial community can be disturbed giving rise to dysbiosis that predisposes the site to disease [3]. Dysbiotic subgingival communities are characterized by a relative increase in abundance of anaerobic, Gram-negative bacterial species that can exploit protein-rich GCF as a nutrient source, promoting development of a proteolytic community phenotype. While around 60% of individuals are estimated to have periodontal pockets at occasional sites in the dentition, only around 7% will develop such severe disease that they risk losing teeth [4, 5]. Currently, the lack of robust tools to identify the individuals with a high degree of risk for tooth loss means that even people who may not develop severe disease undergo intensive treatment, placing a significant burden on healthcare budgets in the developed world.

The subgingival microbiota in the periodontal pocket comprises thin, densely packed biofilms associated with the tooth and epithelial lining, and a community of loosely attached “planktonic cells” in the bulk fluid [6]. In GCF, survival most likely depends on co-operation between different members of the microbial community since a wide range of different catabolic enzymes is required to exploit the complex proteins and glycoproteins as a nutrient source [7]. In addition to metabolic cross-talk, the microorganisms participate in synergistic and antagonistic interactions which determine the overall properties and contribute to the resilience of the microbial community in the face of environmental fluctuations [8, 9]. Many studies have focused on the composition of sub-gingival microbial communities in periodontitis and comparison between healthy subjects and patients with periodontitis has revealed a strong association between Gram-negative, proteolytic species such as *Treponema denticola*, *Tannerella forsythia* and *Porphyromonas gingivalis* (termed red-complex organisms) as well as a less stringent association between *Prevotella* spp., *Fusobacterium* spp. and *Parvimonas micra* (termed orange-complex organisms) and increasing depth of the periodontal pocket [10]. Involvement of these organisms is supported by 16S *r*RNA sequencing studies where *Treponema* spp., *T. forsythia*, *P. gingivalis*, *P. micra, Streptococcus* spp. and *Peptostreptococcus* spp. showed a higher prevalence and abundance in patients with periodontitis than in healthy individuals [11].

The role of periodontitis-associated microorganisms in dysbiosis has largely focused on *P. gingivalis,* revealing this organism to be capable of compromising the host immune response through impairment of leukocyte activity and cytokine paralysis. This has led to the proposal of *P. gingivalis* as a keystone pathogen in the disease [12, 13]. However, while inoculation of specific pathogen-free mice with *P. gingivalis* induces significant periodontal bone loss, this is not the case in germ-free animals, indicating that the capacity of this organism to cause disease is dependent upon the presence of other bacteria [14]. Thus, it appears likely that interactions between the members of the periodontal microbial community could influence the virulence of *P. gingivalis* and the composition of the community surrounding *P. gingivalis *in situ could explain why, for instance, the levels of gingipains in the periodontal pocket vary between patients [15].

In this study, we characterize the virulence properties of *P. gingivalis* strains in a serum environment, both alone and as part of a model subgingival polymicrobial community with three other important members of the periodontitis core microbiome. We show that polymicrobial interactions play a central role in the development of a community-wide virulent phenotype (proteolytic activity) related to gingipain activity from *P. gingivalis*.

## Methods

### Bacterial strains

The *P. gingivalis* strains used were ATCC 53978 (W50) [16] as well as two clinical strains (33F and SUB1) freshly isolated from sub-gingival pocket samples of patients with periodontal disease [> 2 sites with inflammation (bleeding on probing) and alveolar bone loss exceeding 1/3 root length] [17]. After recovery on Brucella agar, clinical strains were identified as *P. gingivalis* through morphology (glossy black-pigmented colonies containing small Gram-negative coccoid rods) and physiological testing (positive for trypsin and indole production but negative for fluorescence and β-galactosidase activity). Isogenic mutants of W50 (E8—expressing only Kgp and K1A—expressing only Rgp) were used to study the role of gingipains in growth of the multi-species communities. The *Parvimonas micra* strain (ECE) was recovered as small white colonies on Brucella agar from a sub-gingival pocket sample of a patient with periodontal disease (see above) while *F. nucleatum* (BK:0) was recovered from sub-gingival plaque of a healthy individual. The identities of the clinical strains of *P. gingivalis*, *P. micra* and *F. nucleatum* were confirmed by 16S *r*RNA gene sequencing. *Streptococcus constellatus* was a reference strain (NCTC 10,714). All strains were stored at − 70 °C in skim milk (Oxoid) and routinely cultured on Brucella agar at 37 °C under anaerobic conditions (10% H_2_, 5% CO_2_ in N_2_).

### Growth of individual strains

The *P. gingivalis* strains were grown in pre-reduced nutrient broth [Bacto™ brain–heart infusion supplemented with 500 µg/mL L-cysteine (BHI)] for 4 days under anaerobic conditions with equal numbers of bacteria [absorbance at 600 nm (A_600_) = 0.1] in the starter cultures. Aliquots were removed at intervals and bacterial growth assessed as increase in A_600_. The capacity of the *P. gingivalis* strains as well as *P. micra*, *F. nucleatum* and *S. constellatus* to grow in a serum environment was evaluated by inoculating them into heat-inactivated equine serum (Håtunalab, AB, Bro Sweden) diluted 1:5 with PBS to give A_600_ = 0.1 and maintaining under anaerobic conditions. Aliquots were removed at intervals and bacterial growth assessed as above.

### Measurement of proteolytic activity

Proteolytic activity was determined by mixing 10 µL aliquots of the bacterial suspensions with 100 µL FITC-conjugated gelatin (1 mg/mL DQ™ gelatin, Molecular probes) in a 96-well plate and incubating at 37 °C. Fluorescence was measured at 1 min intervals using a BMG Clariostar plate reader [excitation 485 nm, emission 530 nm). Values for the negative control (BHI broth or serum alone) were subtracted, compared with a standard curve constructed using trypsin, and the final values expressed as trypsin equivalent units  per min (TEU/min). To assess cell-associated proteolytic activity, a 10 µL aliquot of bacterial suspension was incubated with 1 µL FITC-conjugated gelatin for 30 min at 37 °C and then viewed using a Nikon Eclipse TE2000 inverted confocal scanning laser microscope (CSLM) (Nikon Corp., Tokyo, Japan). Images were acquired with an oil immersion objective (× 60) and illumination provided by an Ar laser (488 nm excitation).

### Zymography

Cell suspensions containing approximately 20 µg of protein were run at 125 V, 4 °C on Novex 10% Zymogram plus gels containing gelatin. The running buffer [25 mM Tris, 192 mM glycine, buffer pH 8.3 containing 0.5% SDS] was kept at 4 °C. Gels were renatured by replacement of SDS with 25% Triton X-100 using Novex Zymogram Renaturing buffer (4 °C for 30 min) so that proteins were shifted from non-catalytic to catalytic conditions. Gels were equilibrated with Zymogram Developing Buffer containing 2 mM L-cysteine for 30 min at 4 °C. Fresh developing buffer containing 2 mM L-cysteine was then added and the gel incubated at 37 °C for 2 h. Finally, gels were stained with colloidal Coomassie brilliant blue G overnight and excess stain removed in 25% ethanol for 1 h at room temperature. All gels and reagents were from Invitrogen. Preparations of Kgp (GingisKHAN®) and Rgp (Gingis REX®) purchased from Genovis AB, Sweden were used for comparison.

### Two-dimensional gel electrophoresis

Late log-phase cultures of the *P. gingivalis* strains were centrifuged (3000*g*, 15 min, 4 °C), and the supernatants filtered to remove bacterial cells. Filtrates were mixed 1:10 with ice-cold TCA and maintained overnight on ice. Precipitated extracellular proteins were harvested by centrifugation (16,000*g*, 30 min, 4 °C) and the pellets re-suspended in ice-cold acetone, sonicated (3 × 10 s) and subjected to a second round of centrifugation. After removal of the acetone, the pellet was air-dried, re-suspended in 1 mL rehydration buffer (8 M urea, 2% CHAPS, 10 mM DTT, 2% IPG buffer; GE Healthcare Life Sciences) and stored at − 20 °C until use. The protein concentration was determined using a 2D-Quant kit (GE Healthcare Life Sciences). Volumes corresponding to 20 μg protein were subjected to 2D-gel electrophoresis essentially as described previously [18]. Isoelectric focusing was carried out on 18-cm pH 4–7 linear immobilized pharmalyte gradient strips followed by gel electrophoresis on 10% polyacrylamide gels. Gels were stained with colloidal Coomassie brilliant blue G. The most abundant proteins were excised manually and subjected to LC–MS/MS as described previously [32]. Mass lists were created automatically and used as the input for Mascot MS/MS Ions searches of the NCBInr database using the Matrix Science web server. Parameters were set to 0.5 Da peptide mass tolerance, methionine oxidation and carboxyamidomethyl cysteine modification.

### Adherence assay

Mini flow-cells (IbiTreat µ-slide VI) were coated overnight at room temperature with serum diluted 1:5 in PBS (0.15 M NaCl, 10 mM NaH_2_PO_4_ buffer, pH 7.4) and the channels rinsed twice with pre-reduced PBS before use. Suspensions of the *P. gingivalis* strains were prepared by harvesting colonies from Brucella agar plates and dispersing them in pre-reduced PBS to give OD_600_ = 0.4. Aliquots were added to the channels and the slides incubated anaerobically for 2 h. After rinsing 3 times with pre-reduced PBS, adherent bacteria were stained with *Bac*Light Live/Dead stain and viewed with a CSLM as described above. All experiments were carried out in triplicate and data analyzed using GraphPad Prism Software. Bacterial surface coverage on the coated surfaces was compared with that on uncoated ones using a two-tailed Mann Whitney *U*-test and *p* values less than 5% were regarded as significant.

### Formation of polymicrobial communities

Suspensions of each individual bacterium were formed by taking colonies from blood agar and suspending them in serum (1:5 in PBS) to give A_600_ = 0.1. Three different polymicrobial communities were then created by mixing equal volumes of each bacterial suspension (*P. micra*, *S. constellatus* and *F. nucleatum*) with *P. gingivalis* strain W50 or the isogenic mutants; E8 or K1A. Cultures were maintained under anaerobic conditions for up to 9 days and aliquots removed at intervals. The A_600_ and general proteolytic activity (as described above) were monitored. An aliquot was also plated onto Brucella agar and the relative numbers of the different bacteria in the consortium enumerated after identification based on colony morphology [*P. gingivalis*; glossy black-pigmented colonies, *P. micra*; small (1–2 mm) round, peaked colonies showing anaerobic growth but no growth in CO_2_, *S. constellatus*; large (5–10 mm) white colonies containing cocci in chains and *F. nucleatum*; large (6 mm), low convex white/grey glistening colonies containing slender rods with pointed ends]. Differences in CFU at different time points were compared using Student's t-test.

### Real-time quantitative PCR (qPCR)

For qPCR analysis of composition in the polymicrobial communities, DNA was extracted using a QIAamp UCP Pathogen mini-kit (Qiagen) according to the manufacturer’s instructions. A lysozyme treatment step (20 mg/mL lysozyme in 20 mM Tris pH 8.0, 2 mM EDTA, 1,2% Triton X-100), 37 °C for 1 h) and lysis using 0.1 mm glass beads (5 × 5 min × 50 Hz in a Tissue-Lyser LT (Qiagen)] followed by incubation with proteinase K (20 min, 56 °C) were used to ensure adequate extraction from Gram-positive cells. Primer sequences for the 16S *r*RNA genes were: *P. gingivalis* (f:5′-TGTAGATGACTGATGGTGAAAACC-3′, r:5′-ACGTCATCCCCACCTTCCTC-3′) [19], *P. micra* (f:5′-TCGAACGTGATTTTTGTGGAAA-3′, r:5′-GGTAGGTTGCTCACGTGTT ACTCA-3′) [20], *F. nucleatum* (f:5′-CGCAGAAGGTGAAAGTCCTG TAT-3’, r:5'-TGGTCCTCACTGATTCACACAGA-3′) [21] and *S. constellatus* (f:5′-AGATG GACCTGCGTTGT-3′, r:5′-TGCCTCCCGTAGGAGTCT-3′) [22]. Total DNA template used was 4-28 ng in each triplicate reaction and negative controls (no DNA template) were prepared alongside the experimental samples to normalize for background signal in the amplification step. Estimates of cell number were made against standards prepared from tenfold serial dilutions of each bacterium (ranging between 10–18 ng and 1–1.8 pg/µL). Quantifications were based on the following estimated 16S rRNA gene copy numbers and amounts of chromosomal DNA in each cell (*P. gingivalis*: 4 copies, 2.5 fg, P*. micra*: 3 copies, 1.9 fg, *F. nucleatum*: 5 copies, 2.3 fg and *S. constellatus*: 4 copies, 2.4 fg) obtained from the Ribosomal RNA Operon Copy Number Database (https://rrndb.umms.med.umich.edu/search/) and NCBI genome database (http://www.ncbi.nlm.nih.gov/sites/genome) respectively. Differences in cell number at different time points were compared using Student's *t*-test.

### 16S *r*RNA fluorescent in situ hybridization (FISH)

Aliquots from the polymicrobial communities were placed in ibiTreat mini flow-cells and fixed with 4% paraformaldehyde in PBS overnight at 4 °C. FISH was then performed largely as described previously [23]. Briefly, cells were permeabilized using lysozyme (10 mg/mL), washed with ultra-pure water and dehydrated with 50%, 80% and 99% ethanol for 3 min each. Finally, 30 mL of hybridization buffer (0.9 M NaCl, 20 mM Tris–HCl buffer, pH 7.5, with 0.01% sodium dodecyl sulfate and 25% formamide) containing 3.3 pmol/µL of the oligonucleotide probes was added and hybridization performed at 48 ºC for 90 min in a humid chamber. Probes were synthesized by biomers.net Gmbh (Ulm/Donau, Germany). *P. gingivalis* was identified using the POGI probe (CAATACTCGTATCGCCCGTTATTC) [24] fluorescently labeled with Atto-565 (red), *F. nucleatum* was identified using the FUSall307 probe (TCAGTCCCCTTG GCCG) [25] labeled with Atto-488 (green), *P. micra* was identified using the PAMIC1435 probe (TGCGGTTAGATCGGCGGC) [26] double-labeled with Atto-390 (blue) and *S. constellatus* was identified using the STR405 probe (TAGCCGTCCCTTTCTGGT) [27] double-labeled with cyanine3 and ATTO-488 yielding yellow fluorescence. The relative proportion of *P. gingivalis* cells found in clusters as compared to present as solitary cells was investigated by manually counting FISH-labelled cells in images obtained using an inverted confocal scanning laser microscope (see above). Four representative images from each of 3 independent experiments were analysed and the mean percentage of solitary cells calculated using GraphPad Prism Software.

## Results

### *Porphyromonas gingivalis* strains show different rates of growth and proteolytic activities

**Fig. 1:**
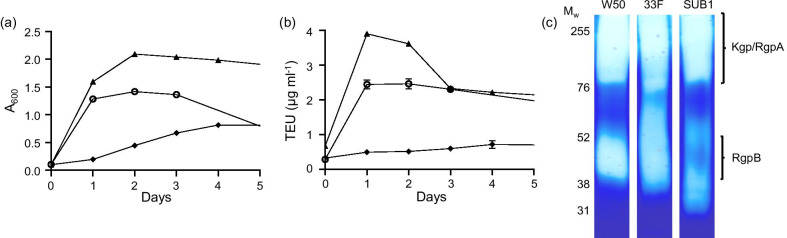
Growth and proteolytic activity of *P. gingivalis* strains in nutrient-rich broth. **a** Growth of strains [33F (filled triangle), W50 (open circle) and SUB1 (filled diamond)] in BHI over time was assessed as absorbance at 600 nm and the graph shows mean ± SD of three independent biological replicates. **b** Proteolytic activity over time as assessed using a FITC-conjugated gelatin substrate and expressed as trypsin equivalent units (TEU). The graph shows mean ± SD of the same three independent biological replicates in **a**. **c** Aliquots from the cultures on day 3 were subjected to zymography on gelatin-containing gels stained with Coomassie brilliant blue. All samples were run on the same gel and the image has not been subject to digital enhancement or bands removed by cropping. The original image is available in Additional file 1

Initially, growth of *P. gingivalis* strains (W50, 33F or SUB1) was compared under anaerobic conditions in nutrient broth for 5 days. Their growth rates differed significantly, with W50 and 33F showing rapid and strong growth over the first 48 h while SUB1 showed low initial growth which continued to increase steadily over time (Fig. 1a). Since extracellular proteases are important in the generation of nutrients for cell growth, we investigated whether growth rate was related to the proteolytic activity in the cultures. On days 1 and 3, strain 33F demonstrated a higher level of proteolytic activity than W50, while SUB1 showed a lower level (Fig. 1b), indicating that the relative level of proteolytic activity corresponded well to the observed growth profile for each strain. Zymography of culture supernatants demonstrated a similar pattern for the three strains, with 2 prominent clear areas; one at a high-M*r* (> 76 kDa) and a second in the region below 60 kDa (Fig. 1c). Comparison with commercial preparations of Kgp and Rgp revealed the high and low-M*r* bands to coincide with Kgp/RgpA and RgpB, respectively. Although only semi-quantitative, the gels appeared to indicate that Kgp/RgpA activity was similar for all strains whereas the RgpB band was most intense for strain 33F and least intense for SUB1.

### Identification of extracellular proteases of *P. gingivalis* strains

**Fig. 2.**
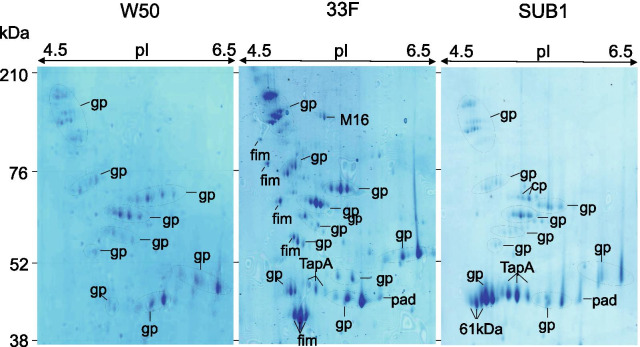
2DE gels of extracellular proteins from *P. gingivalis* strains. Supernatants from cultures of *P. gingivalis* strains W50, 33F and SUB1 were subjected to 2D-PAGE and the gels stained with Coomassie brilliant blue. The major spots were excised and proteins identified using LC–MS/MS. Protein identities are presented in Table 1

To further investigate the proteins associated with activity in the zymograms, supernatants from the *P. gingivalis* cultures were subjected to 2D-PAGE, stained with Coomassie blue and the major spots identified using LC–MS/MS. The protein profiles of the three strains shared some common features in the high-M*r* range (> 76 kDa) but more variation was seen in the spots present at lower M*r* (Fig. 2). Mapping of the peptide sequences from the most intense spots to *P. gingivalis* genomes revealed the presence of six major proteins, which could be clustered into different functional groups (Table 1). Strain 33F showed multiple spots identified as fimbrillin as well as the outer membrane protein Tap A, which was also highly expressed in SUB1. Neither of these were seen in W50. Peptidyl arginine deiminase was also present in strain 33F but not SUB1 or W50. All three strains gave rise to multiple spots corresponding to known *P. gingivalis* proteases, predominantly various forms of Rgp and Kgp. Due to the high degree of sequence homology between these two molecules, it was not always possible to differentiate between them with certainty and spots are therefore annotated as gingipain. The most prominent spots in the high-M*r* range (around 200 kDa) corresponded to full-length versions of gingipains. Additional spots at lower molecular weights were also identified as gingipains. Two other proteases; a member of the M16 protease family and a conserved hypothetical protein with a zinc carboxypeptidase domain, were identified in one spot in 33F and SUB1 respectively. Taken together, these data suggest that gingipains most likely account for most of the extracellular proteolytic activity in all three *P. gingivalis* strains in nutrient broth.

**Table 1:** The most abundant proteins in the exoproteome of different strains of *P. gingivalis* identified using 2D-gel electrophoresis and MS/MS-LC mass spectroscopy

Symbol	Protein description	*Porphyromonas gingivalis* strains		
		W50	33F	SUB1
gp	Gingipain	+ + +	+ + +	+ + +
fim	Fimbrillin	−	+ +	−
M16	Peptidase M16 family	−	+	−
TapA	CTD-containing outer membrane protein	−	+ +	+ + +
pad	Peptidylarginine deiminase	−	+	+
cp	Zinc carboxypeptidase	−	+	−

Mass lists were used as the input for Mascot MS/MS Ions searches of the NCBInr database using the Matrix Science web server

### Survival of P. gingivalis alone in a serum environment

**Fig. 3:**
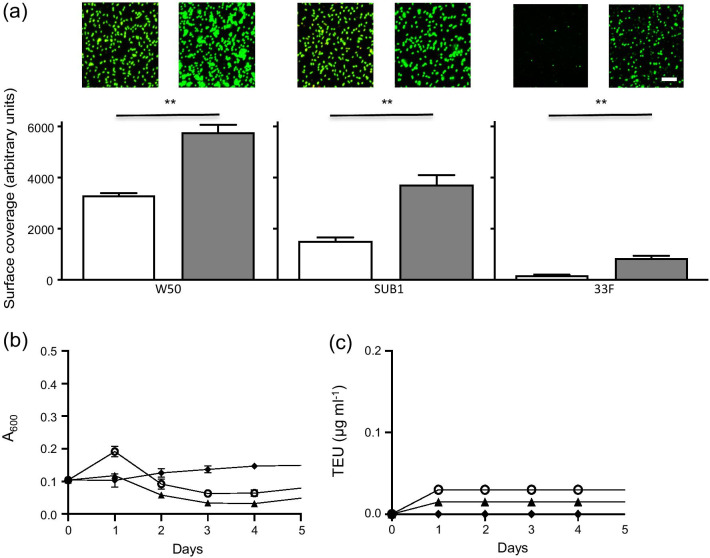
Survival of *P. gingivalis* strains in a serum environment. **a** Graphs showing coverage of *P. gingivalis* strains 33F, W50 and SUB1 on surfaces in the presence (filled square) or absence (open square) of a serum coating. The bars show the mean ± SD of three independent biological replicates. Representative images show adherent bacteria stained with Baclight Live/Dead stain. The bar represents 10 µm. **p < 0.01. **b** Growth of *P. gingivalis* strains 33F (filled triangle), W50 (open circle) and SUB1 (filled diamond) in serum, assessed as absorbance at 600 nm. **c** Graph showing proteolytic activity of *P. gingivalis* strains W50 (open circle), K1A (open triangle) or E8 (filled circle) in serum. The graph shows mean ± SD of three independent biological replicates

The ability to attach to a surface is an important virulence factor for *P. gingivalis* in colonizing the periodontal pocket. Therefore, the different *P. gingivalis* strains were tested for their ability to form biofilms on serum-coated surfaces (Fig. 3a). CSLM revealed significantly higher coverage (1.75–4-fold increase) on the serum-coated surface for all strains compared to an uncoated surface (*p* < 0.01). The absolute levels of coverage on the serum-coated surfaces differed with W50 showing a significantly higher capacity for adherence than the clinical isolates (SUB1 and 33F). However, all strains formed large aggregates in solution suggesting that they interact with proteins in serum (data not shown). To determine whether serum could support growth of the *P. gingivalis* strains, growth of W50, SUB1 and 33F was monitored over 5 days (Fig. 3b). This revealed that none of the strains was able to grow and that this lack of growth was associated with the absence of proteolytic activity in the cultures (Fig. 3c).

### Growth of a multi-species community containing *P. gingivalis* in a serum environment

**Fig. 4:**
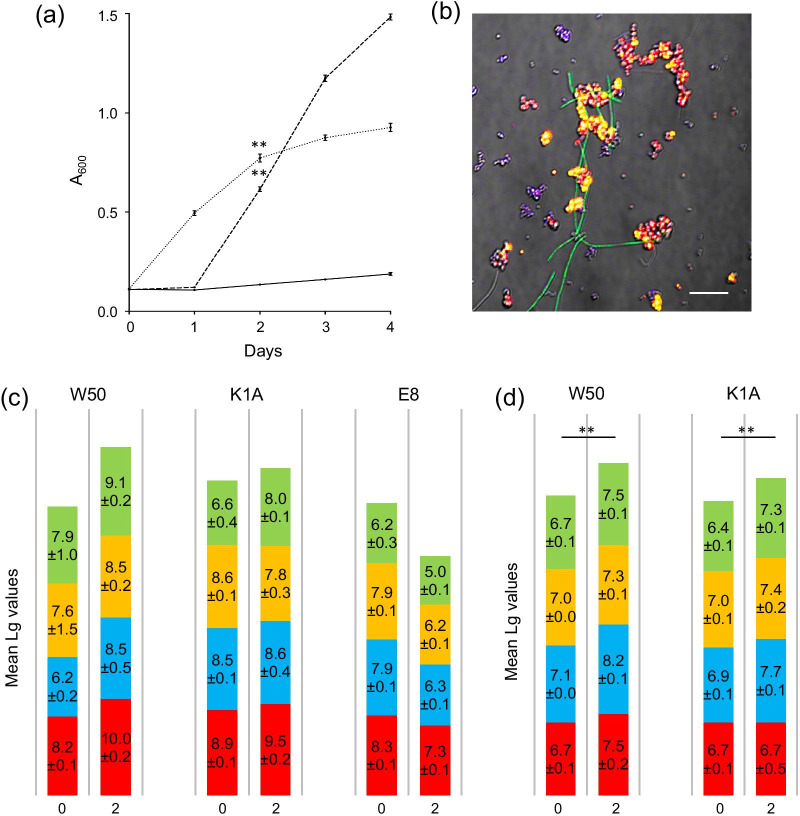
Growth and composition over time of multi-species communities containing *P. gingivalis* strains W50, K1A or E8 in serum. **a** Graph showing growth of multi-species communities containing *P. gingivalis* strain W50 (dotted line), K1A (dashed line) or E8 (solid line) in serum, assessed as absorbance at 600 nm (mean ± SD of three independent biological replicates, **p < 0.01). **b** A representative confocal FISH image showing composition of the W50-containing consortium after 2 days [*P. gingivalis* (red), *F. nucleatum* (green), *P. micra* (blue) and *S. constellatus* (yellow)]. The bar represents 10 µm. Composition of multi-species communities containing *P. gingivalis* strains W50, K1A or E8 on day 0 and day 2 assessed using **c** growth on blood agar or **d** qPCR [*P. gingivalis* (red), *F. nucleatum* (green), *P. micra* (blue) and *S. constellatus* (yellow)]. Numbers represent mean ± SD of three independent replicates, **p < 0.01

The capacity for growth and survival in the serum environment was then tested when *P. gingivalis* was present as part of a multi-species community containing three other sub-gingival colonizers: *F. nucleatum*, *P. micra* and *S. constellatus*. In contrast to mono-species cultures, significant growth of multi-species communities containing *P. gingivalis* strain W50 was seen over 2 days, *p* < 0.01 (Fig. 4a). To ensure that this was not due to the ability of one of the other species to exploit the serum as a growth medium, growth curves were also constructed for *F. nucleatum*, *P. micra* and *S. constellatus* as mono-species cultures. This revealed that none of these bacteria were able to grow alone in serum (data not shown). 16S *r*RNA-FISH revealed the presence of all four bacteria in the multi-species community after 2 days (Fig. 4b). When W50 was replaced in the consortium with the isogenic mutant expressing Rgp only (K1A), significant growth was again facilitated over 2 days whereas no consortium growth was seen when the Kgp expressing mutant was included (Fig. 4a).

A more detailed compositional analysis of the communities containing *P. gingivalis* strains W50, K1A and E8 was then undertaken using culture techniques (Fig. 4c). For the consortium with W50 at time 0, the bacterial count was around 10^8^ cells/mL, (corresponding to OD_600_ = 0.1), with an approximately equal distribution of the four species. After 2 days, there was a significant increase in total counts to approximately 10^10^ cells/mL (corresponding to OD_600_ = 0.6, *p* < 0.01). This growth could be attributed to significant increases in *P. gingivalis* (*p* < 0.01), *F. nucleatum* (*p* < 0.01) and *S. constellatus* (*p* < 0.01) whereas the increase in *P. micra* did not reach significant levels. A similar pattern was seen for the consortium containing K1A with a significant increase in OD_600_ from 0.1 to 0.7, corresponding to 10^8^ and 3 × 10^9^ cells/mL, respectively. Again, this increase was explained by significant increases in *P. gingivalis* (*p* < 0.05), *F. nucleatum* (*p* < 0.01) and *S. constellatus* (*p* < 0.01) whereas the increase in *P. micra* did not reach significant levels. In contrast, the consortium containing E8 showed no growth over 2 days (no change in OD_600_) and the number of living bacteria decreased from around 10^8^ to 10^7^ cells/mL. For the W50 and K1A-containing consortia that showed growth in serum, the species composition obtained by culturing was investigated using qPCR (Fig. 4d). This showed significant increases in cell number from Day 0 to Day 2 in both the W50-containing (*p* < 0.003) and the K1A-containing (*p* < 0.002) communities, which could be attributed to significant increases in all the species included [(W50-containing community; *P. gingivalis* *p* < 0.05, *P. micra* *p* < 0.005, *F. nucleatum* *p* < 0.01, *S. constellatus* *p* < 0.05) and (K1A-containing community *P. gingivalis* *p* < 0.05, *P. micra* *p* < 0.005, *F. nucleatum* *p* < 0.001, *S. constellatus* *p* < 0.01)].

### Development of proteolytic activity in the multi-species community

**Fig. 5:**
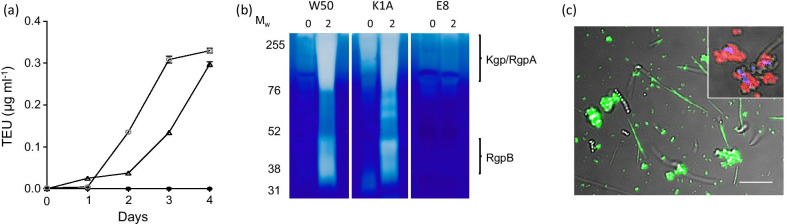
Proteolytic activity in multi-species communities containing *P. gingivalis* strains W50, K1A or E8 in serum. **a** Graph showing proteolytic activity of the multi-species communities containing *P. gingivalis* strains W50 (open circle), K1A (open triangle) or E8 (filled circle) in serum. The graph shows mean ± SD of three independent biological replicates. **b** Aliquots (2 µL) from the cultures on day 0 and day 2 were subjected to zymography on gelatin-containing gels stained with Coomassie brilliant blue. All samples were run on the same gel and the image has not been subject to digital enhancement or bands removed by cropping. The original image is available as Additional file 2. **c** A representative image of bacteria from the multi-species community incubated with FITC-gelatin (green). The insert is a confocal 16S *r*RNA-FISH image showing the presence of *P. gingivalis* (red) and *P. micra* (blue) within the proteolytically-active bacterial clusters. The bar represents 10 µm. Image analysis revealed that 96 ± 1.9% of *P. gingivalis* cells were present in these clusters

Growth of the consortium containing W50 and K1A but not E8 strongly suggests a role for Rgp in facilitating community growth in serum. We therefore investigated development of proteolytic activity in the multi-species community containing *P. gingivalis* strain W50 and the isogenic mutant strains over time. This showed that the total activity increased significantly over 4 days in the communities containing W50 and K1A, but not E8 (Fig. 5a). Zymogram analysis revealed bands associated with the positions of Kgp/RgpA and RgpB in the W50-containing consortium (Fig. 5b). Similar bands were seen in the consortium containing the Rgp-expressing strain, but not that expressing Kgp, suggesting that the activity in the W50 consortium can mainly be attributed to Rgp. Application of the fluorescent general protease substrate to bacteria in the multi-species community revealed that the strongest activity was associated with clumps of cells (Fig. 5c). The 16S *r*RNA-FISH images revealed these proteolytic clusters to correspond to co-aggregates of *P. gingivalis* and *P. micra* (Fig. 5c, insert) and image analysis of around 10,000 cells in each of 3 independent experiments revealed that around 94 ± 1.9% of the *P. gingivalis* population were present in these clusters. Chains of *S. constellatus* were unstained, confirming that they did not express proteolytic activity on the cell surface, however some weak staining was seen on long spindle-shaped *F. nucleatum* cells (Fig. 5c). However, *F. nucleatum*, *S. constellatus* and *P. micra* did not show any proteolytic activity alone in either nutrient-rich broth or serum as shown by zymography (data not shown). Together, these data suggest that Rgp from *P. gingivalis* is the major component of the overall proteolytic phenotype of the community and is essential for growth of all community members.

## Discussion

It is widely acknowledged that periodontitis is associated with a substantial enrichment of anaerobic and proteolytic bacteria in sub-gingival microbial communities. The driving force for this dysbiosis is thought to be increased flow of protein-rich GCF, but the underlying mechanisms are not fully understood. We therefore investigated development of proteolytic activity in a multi-species community, a factor that we propose is essential to the initiation and progression of periodontitis. Although *P. gingivalis* belongs to the red complex as defined by Socransky et al. [10] this organism alone is not sufficient to cause disease. To investigate the role of community interactions in the development of virulence properties in *P. gingivalis*, three other bacteria found in the orange complex as well as in the core microbiome in periodontitis and thereby known to co-exist with *P. gingivalis *in vivo* (P. micra, F. nucleatum* and *S. constellatus),* were included in the model. Previous studies have indicated that *P. micra* and *F. nucleatum* can interact in biofilms [28] while peptides from oral streptococci can affect gingipain expression in *P. gingivalis* [29].

Prior to studies in the community, the virulence properties (ability to bind to serum-coated surfaces, growth and proteolytic activity) of three *P. gingivalis* strains were investigated. Serum increased surface coverage of all strains, suggesting that proteins in the gingival exudate enhance adherence of *P. gingivalis,* thus promoting colonization of the periodontal pocket. Proteomic analysis revealed multiple extracellular isoforms of fimbrillin in strain 33F, consistent with expression of FimA fimbriae, whereas no fimbrillin was seen in strains SUB1 or W50, in keeping with reports that W50 is sparsely fimbriated [30, 31]. Strain 33F showed the lowest absolute level of binding and the lack of a strong relationship between the presence of fimbrillin and adherence suggests that FimA is not critical for binding to surface-associated serum proteins. This is in agreement with a previous study showing that *P gingivalis* fimbriae do not bind to serum albumin [32] although FimA fimbriae are known to be important for binding of *P. gingivalis* to other bacteria such as *Streptococcus oralis* [33].

Consistent with previous data [34], the growth rates in nutrient-rich broth differed between the *P. gingivalis* strains and this was clearly related to the level of proteolytic activity in the cultures. Zymography revealed the secreted proteolytic activity to be present as 2 major bands consistent with the predicted positions of RgpA/Kgp and RgpB respectively [35]. Characterization of extracellular proteins confirmed that gingipains, secreted via the type IX secretion system [36], were the predominant proteases from all the wild-type *P. gingivalis* strains in nutrient broth. As well as the high-M_*r*_ forms of Rgp and Kgp, a large number of lower M_*r*_ forms of gingipain, most likely corresponding to fragments, were identified. The broad bands seen on the zymogram gels appear to indicate that many were proteolytically active. The clinical strains (33F and SUB1), produced two metalloproteinases: a zinc-carboxypeptidase and an M16-family protease, whose functions are currently unknown, as well as an outer membrane protein; Tap A (61KDa antigen) belonging to the CTD protein family, thought to be involved in *P. gingivalis* virulence [37], and peptidylarginine deiminase, involved in protein citrullination. None of these was found in W50. Overall, the extracellular proteomes agree with those seen in other studies and, in keeping with previous work, there are variations between the different strains which may impact on their virulence as well as their ability to survive in multi-species communities [38].

The *P. gingivalis* strains used in this study were unable to grow as mono-species cultures in serum. Previous investigations of the ability of *P. gingivalis* to exploit serum proteins show conflicting results, with some suggesting that *e.g.* human or bovine serum albumin can support growth and others suggesting that it does not [39–41]. However, importantly, all these studies used defined basal media supplemented with serum proteins rather than serum alone. Introduction of *P. gingivalis* into the multi-species consortium containing *S. constellatus*, *P. micra* and *F. nucleatum* led to steady growth. Community composition remained stable over time as shown using culturing techniques and qPCR, indicating that all four species contributed to community growth. This suggests that coordinated degradation, through expression of the required repertoire of enzymes, allowed the community to exploit the complex proteins and glycoproteins in serum which could not be utilized by any of the individual species alone. Similar phenomena have been described previously, where almost complete degradation of serum proteins was achieved by a plaque-derived consortium selected through growth in serum [42, 43] and a consortium of plaque bacteria was required to degrade complex salivary glycoproteins [44]. In parallel with growth, the overall level of proteolytic activity in the community increased. The role of gingipains in community growth was investigated by replacing W50 with the isogenic mutant strains; K1A expressing only Rgp or E8 expressing only Kgp. In the presence of K1A, culturing and qPCR revealed growth of all the community species, whereas no growth was seen in the community containing E8. This suggests that Rgp from *P. gingivalis* plays a central role in facilitating growth and maintaining diversity of the whole community through the provision of nutrients, mainly peptides and amino acids, for growth. This was confirmed by zymography which revealed a prominent band corresponding to RgpB in the consortia containing W50 and K1A, which was absent in the E8-containing consortium. Confocal microscopy with FISH and the fluorescent proteolytic substrate revealed that in the consortium, *P. gingivalis* were rarely present as solitary cells (6 ± 0.9%) and the majority were found in strongly proteolytic clusters with *P. micra*. In addition, some activity was associated with long, spindle-shaped cells of *F. nucleatum*, but it was not possible to determine whether this was due to expression of serine proteases known to be produced by this species [45] or, for instance, sequestration of gingipains onto the *F. nucleatum* cell surface. Thus, we have shown that growth of all species in the multi-species community appears to be dependent on the activity of RgpB from *P. gingivalis*. While W50 expressed high levels of Kgp/RgpA and RgpB in nutrient-rich broth, this wild type strain showed no growth or proteolytic activity in single-species culture in serum suggesting that gingipain expression in this environment required the presence of other members of the consortium. Gingipain expression has been proposed to be regulated by environmental cues and nutritional status [33] and we have shown previously that Rgp expression increases significantly in multi-species communities in response to heat-sensitive factors from *P. micra* [46]. This could explain why *P. gingivalis* requires the presence of the commensal microflora for the development of periodontitis in animal models [14] as well as the observation that *P. gingivalis* is often isolated from apical periodontal abscesses as part of a consortium together with *Fusobacterium* and *P. micra* [47].

## Conclusions

*Porphyromonas gingivalis* strains grow at different rates and show different levels of proteolytic activity in nutrient broth, but despite being able to attach to serum-coated surfaces, none of the strains used here was able to survive and grow alone in a serum environment. In a multi-species consortium, community growth was facilitated in the presence of wild-type and an Rgp-expressing strain of *P. gingivalis* suggesting that Rgp is involved in delivery of nutrients through degradation of complex serum substrates. Whereas they are constitutively expressed by *P. gingivalis* in nutrient broth, Rgp expression in an environment modelling the periodontal pocket appears to be orchestrated through signaling to *P. gingivalis* from other members of the community. This phenomenon facilitates growth of the whole community thus likely contributing to the maintenance of microbial diversity within the periodontal pocket.

## Supplementary Information

**Additional file 1**. Zymogram gel showing aliquots (2μl) from 3-day old cultures of* P. gingivalis* strains [W50, 33F, SUB1 and 2 strains not otherwise mentioned in the paper (ATCC33277 and 16A) as well as commercial preparations of Kgp and RgpB] on a gelatin-containing gel stained with Coomassie brilliant blue. This image has not been subjected to digital enhancement.

**Additional file 2**. Zymogram gel showing aliquots (2μl) from day 0 and day 2 cultures of* P. gingivalis* strains W50, E8 and K1A on a gelatin-containing gel stained with Coomassie brilliant blue. This image has not been subjected to digital enhancement.

## Data Availability

The datasets used and/or analysed during the current study are available from the corresponding author on reasonable request.
