# Supplementary material for: Polymicrobial synergy stimulates Porphyromonas gingivalis survival and gingipain expression in a multi-species subgingival community
Source: BMC Oral Health. 2021 Dec 15;21:639. doi: 10.1186/s12903-021-01971-9 (PMC8672593; doi:10.1186/s12903-021-01971-9)
Supplement: Supplementary file 1 — Additional file 1. Zymogram gel showing aliquots (2μl) from 3-day old cultures of P. gingivalis strains [W50, 33F, SUB1 and 2 strains not otherwise mentioned in the paper (ATCC33277 and 16A) as well as commercial preparations of Kgp and RgpB] on a gelatin-containing gel stained with Coomassie brilliant blue. This image has not been subjected to digital enhancement. [file 12903_2021_1971_MOESM1_ESM.pptx]

## Slide 1
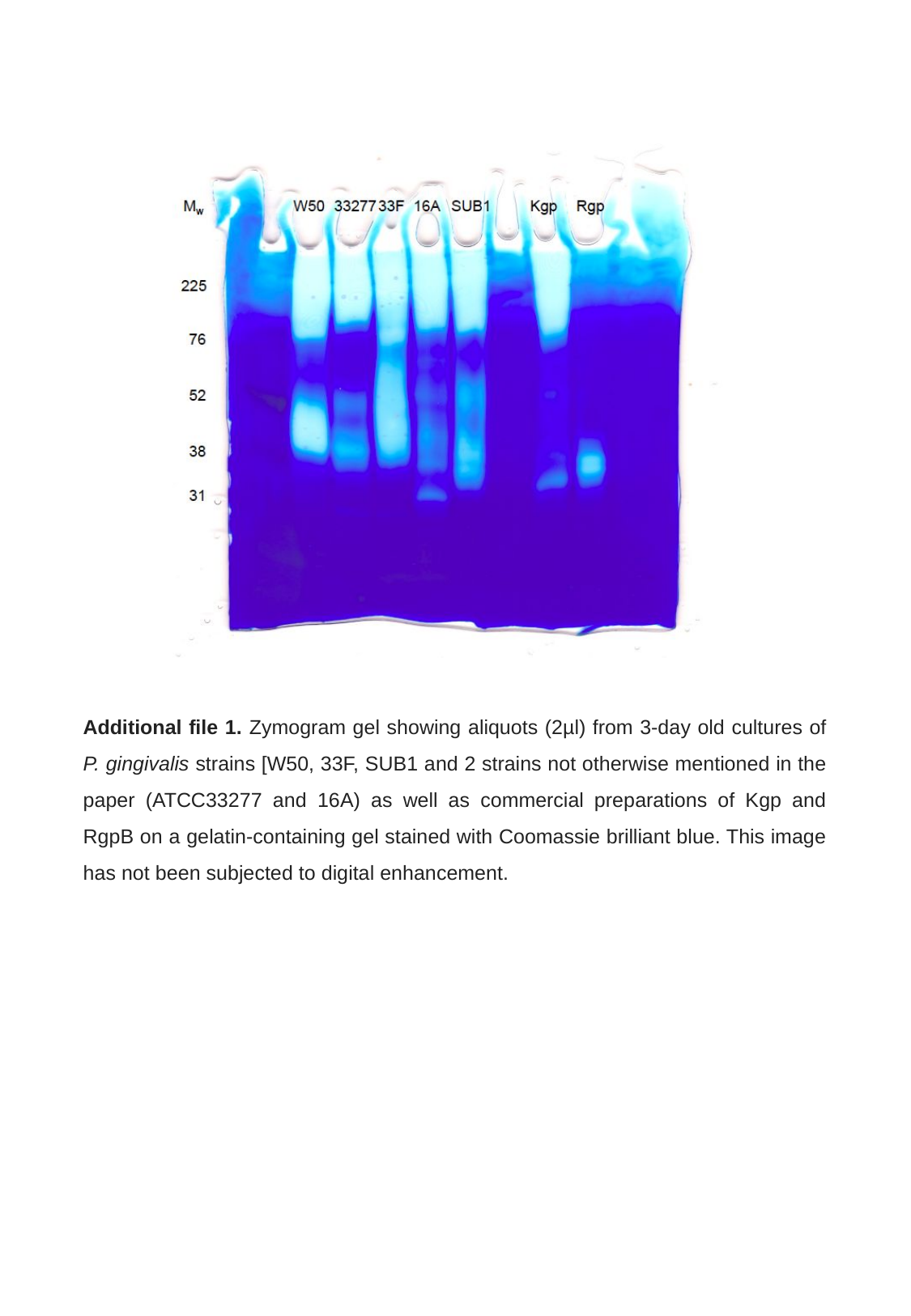

Additional file 1. Zymogram gel showing aliquots (2µl) from 3-day old cultures of P. gingivalis strains [W50, 33F, SUB1 and 2 strains not otherwise mentioned in the paper (ATCC33277 and 16A) as well as commercial preparations of Kgp and RgpB on a gelatin-containing gel stained with Coomassie brilliant blue. This image has not been subjected to digital enhancement.
