# Supplementary material for: Polymicrobial synergy stimulates Porphyromonas gingivalis survival and gingipain expression in a multi-species subgingival community
Source: BMC Oral Health. 2021 Dec 15;21:639. doi: 10.1186/s12903-021-01971-9 (PMC8672593; doi:10.1186/s12903-021-01971-9)
Supplement: Supplementary file 2 — Additional file 2. Zymogram gel showing aliquots (2μl) from day 0 and day 2 cultures of P. gingivalis strains W50, E8 and K1A on a gelatin-containing gel stained with Coomassie brilliant blue. This image has not been subjected to digital enhancement. [file 12903_2021_1971_MOESM2_ESM.pptx]

## Slide 1
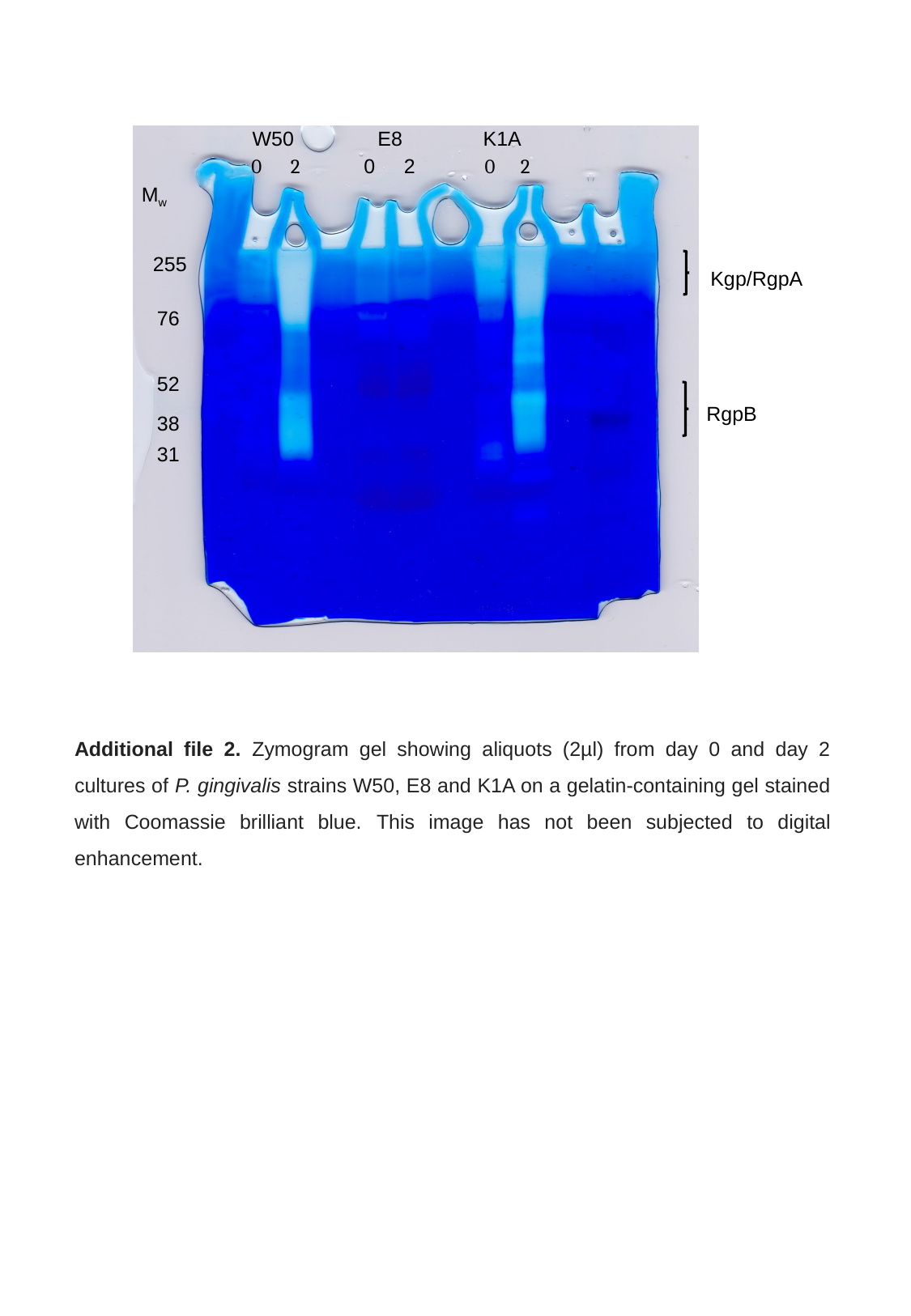

W50
0
2
E8
0
2
K1A
0
2
Mw
255
Kgp/RgpA
76
52
RgpB
38
31
Additional file 2. Zymogram gel showing aliquots (2µl) from day 0 and day 2 cultures of P. gingivalis strains W50, E8 and K1A on a gelatin-containing gel stained with Coomassie brilliant blue. This image has not been subjected to digital enhancement.
